# Do state insurance mandates alter ICSI utilization?

**DOI:** 10.1186/s12958-020-00589-w

**Published:** 2020-04-25

**Authors:** Pavel Zagadailov, David B. Seifer, He Shan, Shvetha M. Zarek, Albert L. Hsu

**Affiliations:** 1 Clinical Outcomes Research Group, CORG LLC, 178 Meadow Brook Rd, Grantham, NH 03753 USA; 2 Department of Obstetrics and Gynecology, Yale University School of Medicine, 333 Cedar St, New Haven, CT 06510 USA; 3 Harvard T.H. Chan School of Public Health, 677 Huntington Ave, Boston, MA 02115 USA; 4 Department of Obstetrics, Gynecology and Women’s Health, University of Missouri School of Medicine, Columbia, MO USA

**Keywords:** Assisted reproductive technology, In-vitro fertilization, Insurance mandates, Intracytoplasmic sperm injection

## Abstract

**Background:**

Assisted reproductive technology (ART) insurance mandates resulted in improved access to infertility treatments like intracytoplasmic sperm injection (ICSI). Our objective was to examine whether ART insurance mandates demonstrate an increased association with ICSI use.

**Methods:**

In this retrospective cohort study, clinic-specific data for 2000–2016 from the Centers for Disease Control (CDC) were grouped by state and subgrouped by the presence and extent of ART state insurance mandates. Mandated (*n* = 8) and non-mandated (*n* = 22) states were compared for ICSI use and male factor (MF) infertility in fresh non-donor ART cycles with a transfer in women < 35 years. Clinical pregnancy (CPR), live birth (LBR) rates, preimplantation genetic testing (PGT), elective single-embryo transfer (eSET) and twin birth rates per clinic were evaluated utilizing Welch’s t-test. Pearson correlation was used to measure the strength of association between MF and ICSI; ICSI and CPR, and ICSI and LBR over time. Results were considered statistically significant at a *p*-value of < 0.05, with Bonferroni correction used for multiple comparisons.

**Results:**

From 2000 to 2016, ICSI use per clinic increased in both mandated and non-mandated states. ICSI use per clinic in non-mandated states was significantly greater from 2011 to 2016 (*p* < 0.05, all years) than in mandated states. Clinics in mandated states had less MF (30.5 ± 15% vs 36.7 ± 15%; *p* < 0.001), lower CPR (39.8 ± 4% vs 43.4 ± 4%; *p* = 0.02) and lower LBR (33.9 ± 3.5% vs 37.9 ± 3.5%; *p* < 0.05). PGT rates were not significantly different. ICSI use in non-mandated states correlated with MF rates (r = 0.524, *p* = 0.03). A significant correlation between ICSI and CPR (*r* = 0.8, *p* < 0.001) and LBR (*r* = 0.7, *p* < 0.001) was noted in mandated states only. eSET rates were greater and twin rates were lower in mandated compared with non-mandated states.

**Conclusions:**

There was greater use of ICSI per clinic in non-mandated states, which correlated with an increased frequency of MF. In mandated states, lower ICSI rates per clinic were accompanied by a positive correlation with CPR and LBR, as well as a trend for greater eSET rates and lower twin rates, suggesting that state mandates for ART coverage may encourage more selective utilization of laboratory resources.

## Background

State insurance mandates for Assisted Reproductive Technologies (ART, including IVF, or in vitro fertilization) result in increased access to infertility treatment and may influence clinical practice [1]. While seventeen states have partial coverage for some fertility treatments, only eleven states have passed insurance mandates for ART (AR/Arkansas, CT/Connecticut, DE/Delaware, HI/Hawaii, IL/Illinois, MD/Maryland, MA/Massachusetts, NH/New Hampshire, NJ/New Jersey, NY/New York, and RI/Rhode Island) [2–12].

According to the American Society for Reproductive Medicine (ASRM), intracytoplasmic sperm injection (ICSI) is indicated for severe male factor (MF) infertility and for couples with prior failed fertilization with conventional insemination (using in vitro fertilization, or IVF). ICSI for diagnoses like unexplained infertility, low oocyte yield, and advanced maternal age has not demonstrated improved clinical outcomes but is still commonly used in clinical practice [13].

While the percentage of ICSI cycles increased from 1995 (11%) to 2004 (57.5%), MF diagnosis remained constant, suggesting increased use of ICSI for conditions other than MF infertility [14]. Previous studies evaluating state mandates [15] found an association with lower per-cycle use of ICSI for non-male factor indications, especially low oocyte yield and unexplained infertility. However, no previously published study has evaluated ICSI use for MF, using our subgroupings of ART state mandates.

The main objective of this study was to determine whether the presence of state-specific ART insurance mandates influenced the practice of ICSI; we hypothesized that ART insurance mandates would increase ICSI utilization. Based on our previous studies [16, 17], we also evaluated the frequency of MF diagnoses and outcomes of CPR rate per clinic and LBR. A secondary objective was to track how other ART practices (PGT and eSET rates per clinic) and clinical outcomes (such as CPR, LBR and twin birth rates per clinic in women < 35 years of age) have changed over time in these mandates.

## Methods

### Data

This is a population-based, retrospective cohort study evaluating data from the National Assisted Reproductive Technology Surveillance System (NASS), which is maintained by the Centers for Disease Control and Prevention (CDC). Publicly available NASS datasets for 2000–2016 were downloaded from the CDC website [18]. Clinics within each yearly report were grouped by state, according to the presence of state insurance mandates for ART as of 2016. These state groupings were compared for clinic specific ICSI use and frequency of MF diagnosis overall, as well as for CPR and LBR rates, PGT, eSET and twin birth rates per clinic among fresh non-donor ART cycles with a transfer in women < 35 years of age.

Data for Connecticut (CT) and New Jersey (NJ) were included in the analysis at 1 year after their ART mandates were established (2005 for CT, 2001 for NJ). PGT rates were available starting 2007, while eSET from 2008 and twin birth rates were available from 2013 to 2016.

### ART mandated and non-mandated group description

Given that the ART mandates in Delaware (enacted 6/30/18), New York (4/12/19), and New Hampshire (effective on 01/01/20) [4, 11, 12] have only been established recently, we compared the other eight states with mandated ART coverage (AR, CT, HI, IL, MA, MD, NJ, and RI) with a cohort of states with no mandate (*n* = 22: AL, AK, AZ, CO, FL, GA, ID, KS, ME, MI, MN, NE, NV, NM, NC, ND, OR, SC, SD, UT, WA, WY). States that shared any border (*n* = 14: DE, DC, IA, IN, KY, MO, MS, NH, OK, PA, TN, VA, VT, WI) with those eight ART-mandated states were excluded from analysis, as patients at an ART clinic in a “border state” may live or work in a mandated state, and thereby be able to take advantage of mandated ART insurance coverage. Similarly, states with limited mandated insurance coverage for infertility treatment or diagnosis but without specific mandated coverage for ART (*n* = 7: CA, LA, MT, NY, OH, TX, WV) were also excluded from this analysis to highlight the differences between states with ART mandates and states with no specific mandates to cover any infertility diagnosis or treatment. This resulted in a comparison of eight ART-mandated states with 22 non-mandated states. (Table 1).

**Table 1:** Distribution of states by type of mandated insurance coverage for ART

ART-Mandated States	Non-Mandated States	Partial mandates for infertility diagnosis and treatment (excluded from analysis)	“Border states” (excluded from analysis)
Arkansas	Alabama	California	Delaware^d^
Connecticut^a^	Alaska	Louisiana	District of Columbia
Hawaii	Arizona	Montana	Iowa
Illinois	Colorado	New York^c^	Indiana
Maryland	Florida	Ohio	Kentucky
Massachusetts	Georgia	Texas	Missouri
New Jersey^b^	Idaho	West Virginia	Mississippi
Rhode Island	Kansas		New Hampshire^e^
	Maine		Oklahoma
	Michigan		Pennsylvania
	Minnesota		Tennessee
	Nebraska		Virginia
	Nevada		Vermont
	New Mexico		Wisconsin
	North Carolina		
	North Dakota		
	Oregon		
	South Carolina		
	South Dakota		
	Utah		
	Washington		
	Wyoming		

^a^Mandated ART coverage become effective in 2005, data included in the analysis from 2006 to 2016

^b^Mandated ART coverage become effective in 2001, data included in the analysis from 2002 to 2016

^c^A mandate for ART coverage for the State of New York went into effect on February 27, 2019

^d^A mandate for ART coverage for the State of Delaware went into effect on June 30

^e^A mandate for ART coverage for the State of New Hampshire will be effective as of January 1, 2020

### Statistical analysis

Statistical analysis was performed utilizing R (version 3.5.1, R Core Team, University of Auckland, New Zealand). Welch’s t-test was used to test statistical differences between ICSI; MF; CPR and LBR; PGT, eSET and twin rates per clinic between mandated and non-mandated states for ART coverage. Pearson correlation was used to measure the strength of a linear association between rates of MF and ICSI; ICSI and CPR, ICSI and LBR over time. The ratio of ICSI and MF was used to evaluate the role of MF in use of ICSI in mandated and non-mandated states. Results were considered statistically significant at a *p*-value of < 0.05, with Bonferroni correction used for multiple comparisons. Figures are reported as means ± standard deviation as a measure of dispersion.

This study qualified as “not human subject research” by the MU Human Subjects Research Protections Program/IRB at the University of Missouri.

## Results

### Trends analysis

**Fig. 1:**
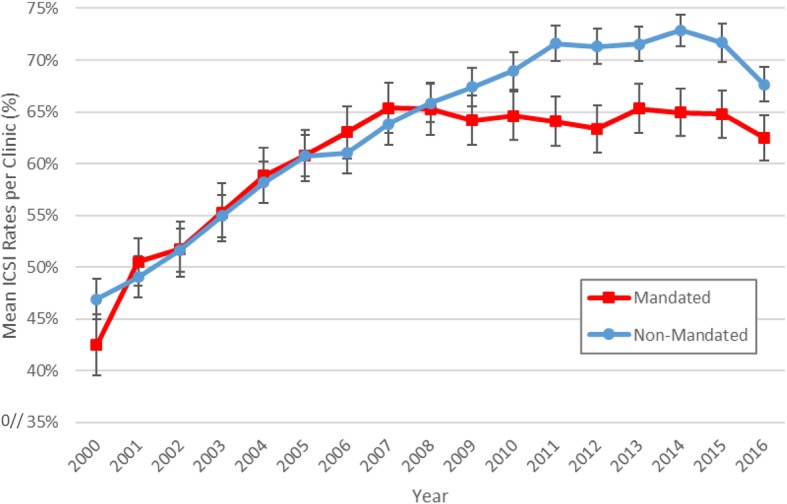
Mean ICSI Rates per Clinic for fresh non-donor ART cycles with a transfer by Type of Mandate from 2000 to 2016

Mean ICSI, MF, PGT, and among women < 35 years of age, CPR and LBR, eSET and twin birth rates per clinic for ART-mandated and non-mandated states are summarized in Table 2. From 2000 to 2016, absolute rates of ICSI use per clinic increased by 20% in both ART-mandated (42.5 ± 20 to 62.5 ± 19%) and non-mandated states (46.9 ± 20 to 67.6 ± 19%). Based on statistical significance, the percentage of ICSI use among clinics in non-mandated states has been greater from 2011 to 2016 (*p* < 0.05) compared with mandated states. (Fig. 1) Male factor diagnoses rate per clinic (Additional file 1) remained at consistent rates in both groups but was greater in non-mandated states (30.5 ± 15% for mandated and 36.7 ± 15% in non-mandated states in 2016) (*p* < 0.001). In contrast, the ratio of ICSI to MF increased over time in both groups and was greater in mandated (1.31 to 2.05) states compared to non-mandated (1.29 to 1.84) states.

**Table 2:** Comparison of ART-Mandated versus the Non-Mandated States from 2000 to 2016

Rates per Clinic, % (MEAN ± STDEV)	2000	2001	2002	2003	2004	2005	2006	2007	2008	2009	2010	2011	2012	2013	2014	2015	2016
Number of Clinics																	
Mandated	44	43	64	62	66	72	75	75	76	78	80	78	78	78	76	78	77
Non-Mandated	110	106	106	109	111	117	121	123	126	123	126	126	123	129	129	132	131
ICSI																	
Mandated	42.5 ± 20	49.5 ± 21	51.7 ± 21	55.3 ± 22	58.9 ± 22	62.1 ± 22	63 ± 22	65.4 ± 21	65.3 ± 22	64.2 ± 21	64.6 ± 21	64.1 ± 21	63.3 ± 20	65.3 ± 21	64.9 ± 20	64.8 ± 20	62.5 ± 19
Non-Mandated	46.9 ± 20	49.1 ± 20	51.7 ± 22	54.9 ± 21	58.2 ± 21	60.8 ± 22	61 ± 22	63.8 ± 22	65.8 ± 21	67.4 ± 20	69 ± 20	71.6 ± 19	71.3 ± 19	71.6 ± 19	72.9 ± 19	71.7 ± 22	67.6 ± 19
*p* value	0.21	0.9	0.98	0.92	0.84	0.57	0.53	0.62	0.99	0.29	0.14	0.01	0.01	0.03	0.01	0.03	0.06
Male Factor (MF)																	
Mandated	32.4 ± 16	33.3 ± 14	33.6 ± 12	32.7 ± 13	35.4 ± 15	34.9 ± 14	33.4 ± 14	35.5 ± 16	34.5 ± 14	33.6 ± 14	33.1 ± 15	32.8 ± 15	34.4 ± 17	34.1 ± 15	32 ± 16	31.3 ± 16	30.5 ± 15
Non-Mandated	36.4 ± 13	37.7 ± 13	37 ± 13	37.8 ± 14	36.9 ± 15	38.5 ± 16	39.3 ± 17	38.4 ± 15	40.2 ± 15	40.7 ± 16	42.1 ± 15	41 ± 14	40.3 ± 16	37.4 ± 15	38.4 ± 16	37.6 ± 15	36.7 ± 15
*p* value	0.08	0.08	0.1	0.02	0.52	0.1	0.01	0.21	0.01	< 0.001	< 0.001	< 0.001	0.01	0.12	0.01	0.01	< 0.001
ICSI/MF Ratio																	
Mandated	1.31	1.49	1.54	1.69	1.66	1.78	1.89	1.84	1.89	1.91	1.95	1.95	1.84	1.91	2.03	2.07	2.05
Non-Mandated	1.29	1.30	1.4	1.45	1.58	1.58	1.55	1.66	1.64	1.66	1.64	1.75	1.77	1.91	1.9	1.91	1.84
Pregn. Rate (< 35)																	
Mandated	29.3 ± 11	35.1 ± 12	37.7 ± 9	38.3 ± 12	39 ± 12	39.1 ± 10	41.6 ± 11	42 ± 10	43.1 ± 10	43.5 ± 11	43.4 ± 14	42.4 ± 13	44.5 ± 11	45 ± 14	40.9 ± 12	37.6 ± 15	34.5 ± 16
Non-Mandated	38 ± 14	40.2 ± 11	42.6 ± 10	43.5 ± 13	41.7 ± 14	43.1 ± 14	44.5 ± 12	46.2 ± 12	47 ± 13	48 ± 13	47 ± 13	46.5 ± 12	46.9 ± 13	46.9 ± 13	43.2 ± 14	36 ± 17	35.6 ± 20
*p* value	< 0.001	0.02	< 0.001	0.01	0.18	0.02	0.08	0.01	0.02	0.01	0.06	0.03	0.15	0.34	0.22	0.49	0.66
Live Birth (< 35)																	
Mandated	25.1 ± 9	30.4 ± 10	31.4 ± 8	32.9 ± 9	32.6 ± 12	33.3 ± 8	35.6 ± 9	36.1 ± 10	36.5 ± 9	37 ± 10	37.2 ± 10	35.7 ± 10	37.6 ± 9	37.5 ± 10	35.8 ± 9	31.9 ± 14	29.2 ± 11
Non-Mandated	33.5 ± 12	35.5 ± 10	37.4 ± 10	38.3 ± 10	36.1 ± 11	37.8 ± 12	39.2 ± 11	40.1 ± 11	40.4 ± 11	42 ± 10	41.4 ± 11	40.6 ± 11	41.3 ± 10	41 ± 11	37.9 ± 12	30.8 ± 15	31 ± 13
*p* value	< 0.001	0.01	< 0.001	0.01	0.06	0.01	0.03	0.01	0.01	< 0.001	0.02	0.01	0.02	0.07	0.22	0.58	< 0.001
PGT Rates	n/a	n/a	n/a	n/a	n/a	n/a	n/a										
Mandated								5.2 ± 9	3.9 ± 8	3.3 ± 5	3.4 ± 5	4.2 ± 7	4.3 ± 6	4.2 ± 6	3.1 ± 6	3.3 ± 6	13.4 ± 13
Non-Mandated								3.2 ± 5	3 ± 5	3 ± 5	3.3 ± 6	4.1 ± 7	4.4 ± 10	5.4 ± 11	4.7 ± 10	4.6 ± 10	21.3 ± 21
*p* value								0.08	0.36	0.74	0.87	0.92	0.92	0.28	0.17	0.28	< 0.001
eSET Rates (< 35)	n/a	n/a	n/a	n/a	n/a	n/a	n/a	n/a									
Mandated									3.1 ± 4	3.6 ± 6	4.4 ± 6	10.1 ± 12	12.2 ± 14	20.4 ± 19	24.7 ± 22	34.5 ± 27	41.5 ± 28
Non-Mandated									2.3 ± 3	3.5 ± 4	4.3 ± 5	8 ± 9	10.9 ± 12	14.9 ± 14	21.3 ± 19	25 ± 23	34.2 ± 28
*p* value									0.15	0.87	0.85	0.19	0.5	0.03	0.27	0.01	0.08
Twin Rates (< 35)	n/a	n/a	n/a	n/a	n/a	n/a	n/a	n/a	n/a	n/a	n/a	n/a	n/a				
Mandated														9.1 ± 5	9.2 ± 6	5.1 ± 4	4.6 ± 2
Non-Mandated														13.2 ± 7	10.5 ± 6	6.8 ± 6	6.2 ± 5
*p* value														< 0.001	0.16	0.02	0.02

From 2000 to 2016, in women < 35 years of age, CPR were generally higher in non-mandated states (38 ± 14 to 35.6 ± 20) compared to mandated (29.3 ± 11 to 34.5 ± 16) states. LBR were somewhat higher in non-mandated states (33.5 ± 12 to 31 ± 13%) compared to mandated (25.1 ± 9 to 29.2 ± 11%) states. Although LBR per clinic were modestly higher in non-mandated states from 2000 to 2011, LBR per clinic were not significantly different from 2012 to 2016 when comparing non-mandated to mandated states.

Additionally, for women < 35 years of age, eSET rates per clinic increased in both groups and were generally greater in ART mandated than non-mandated states, while twin birth rates per clinic decreased in both the ART mandated and non-mandated states but remained greater in non-mandated states. PGT rates were not significantly different between groups.

### Correlation analysis

**Fig. 2:**
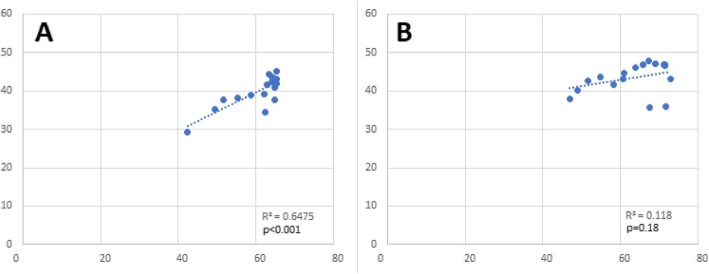
Correlation between ICSI and Clinical Pregnancy Rates per Clinic for fresh non-donor ART cycles with a transfer in Women < 35 Years of Age by Type of Mandate from 2000 to 2016. **a** Mandated States. **b** Non-mandated States

**Fig. 3:**
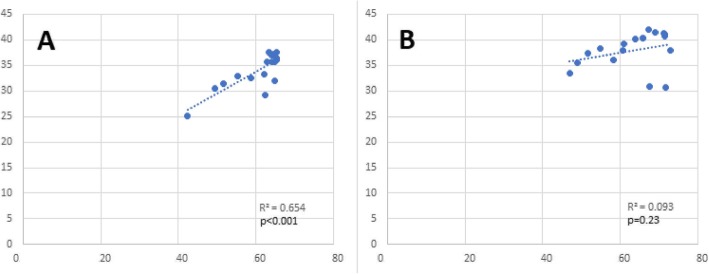
Correlation between ICSI and Live Birth Rates per Clinic for fresh non-donor ART cycles with a transfer in Women < 35 Years of Age by Type of Mandate from 2000 to 2016. **a**. Mandated States. **b**. Non-mandated States

Use of ICSI in non-mandated states correlated with MF rates (*r* = 0.524, *p* = 0.03), while a positive correlation between ICSI and CPR (*r* = 0.8, *p* < 0.001) (Fig. 2) and LBR (*r* = 0.7, *p* < 0.001) (Fig. 3) was observed in ART mandated states only.

## Discussion

This study demonstrates that state insurance mandates for ART are associated with decreased per-clinic ICSI utilization rates in comparison to ICSI use in non-mandated states. In contrast to the cycle-specific findings of Dieke et al. [3] the present study utilizes a different comparison group as well as per-clinic rather than per-cycle rates. After 2011, aggregate ICSI rates per clinic were noted to be lower among the eight ART-mandated states compared to the 22 non-mandated states. Our analysis allowed for more meaningful and informative comparisons of clinic performance as well as regional differences between clinics than prior studies. We were careful to exclude border states out of concern that clinics near state borders may treat women covered by insurance mandates from the neighboring ART mandated state. We also found that per-clinic rates of eSET were higher in ART mandated states, while LBR were not significantly different from 2011 to 2016.

The strengths of this study include the use of data from NASS, which incorporates > 98% of ART cycles performed annually in the U.S. A second strength is in the analysis of clinic-specific rather than cycle-specific rates as in all previous analyses. A comparison of groups of clinics provides a better understanding of variations in ART practice. An additional strength is the exclusion of 14 states that border those eight ART mandated states and the further exclusion of seven states with “partially” mandated coverage for infertility diagnosis and treatment (that does not include coverage for ART). The exclusion of these “border” states is particularly important, because some patients at an ART clinic in a “border” state will live or work in an ART mandated state, and thereby be able to take advantage of that state’s explicit mandate for private insurance companies to cover ART. The exclusion of states with “partial” insurance mandates enables a more precise delineation of the “haves” (states with mandated ART coverage) from the “have nots” (minimal insurance coverage). These specific exclusions allow for a more precise comparison between ART mandated and non-mandated states, unencumbered by confounding variables such as those “border states.”

There are several limitations to our analysis. We were unable to address “partial ICSI” or “ICSI split” cases, in which some oocytes from an ART cycle are inseminated with conventional IVF and other oocytes with ICSI. We were further unable to identify whether IVF or ICSI embryos were transferred in such cases, as such information is not currently available from NASS. We also did not have data on semen parameters, which was also not available from NASS. Additionally, we were not able to evaluate what percentage of ICSI was done specifically in different types of non-male factor infertility cases or to perform a clinic-specific adjustment for a mean number of transferred embryos per cycle, PGT cases and other cycle-specific cofounders, as we have only had access to aggregate clinic-specific data. We limited our study of CPR, LBR, eSET and twin rates to women < 35 years of age because combined overall rates for all ages were not available from the online dataset. Another limitation is that, as with any analysis of large clinical datasets, there is some heterogeneity in data collection and reporting. This was an observational analysis, so we were limited to reporting associations and were unable to make inferences about causality.

A final limitation is that these state ART insurance mandates are heterogeneous. State ART mandates do not cover all patients, as they do not apply to patients working for self-insured employers or to patients who are federal employees with federal health insurance (e.g. TRICARE, Federal Employees Health Benefits, or Medicare/Medicaid). Temporal changes in these mandates is another confounder; for example, Connecticut recently removed their age limit on ART cycles [19]. Massachusetts has the most comprehensive coverage, but a proven infertility diagnosis is first required [20].

The ASRM Practice Committee bulletin on ICSI [13] states that “ICSI is a safe and effective therapy for male factor infertility and couples with prior failed fertilization following conventional IVF.” Furthermore, ICSI may benefit patients undergoing ART with PGT, in vitro maturation (IVM), or with previously cryopreserved oocytes, but the “safety and cost of ICSI in the setting of non-male factor infertility must be considered.” In addition to the procedural costs of ICSI, the medical cost of ICSI may include the increased risks of imprinting disorders and sex-chromosome aneuploidy when insemination of oocytes is accomplished with ICSI rather than IVF [21], as well as other potential neonatal risks of ICSI [22, 23]. ICSI use is low in Western Europe [24], and in contrast to the United States, most Western European countries have socialized medicine and thus have insurance coverage for multiple ART cycles. When patients cannot afford multiple cycles of ART, a physician may be tempted to recommend ICSI to preclude the possibility of an ART cycle with a failed fertilization, given the emotional and financial burden of a failed-fertilization ART cycle for the patient and their families.

There are several clinic and patient-specific reasons for using ICSI. Possible reasons for lower use of ICSI in the eight ART-mandated states include the ability for patients to undergo multiple cycles without incurring high “out of pocket” costs, and possible insurance restrictions on ICSI utilization when not clinically indicated. Furthermore, physicians, as well as embryology laboratory personnel, may prefer ICSI in patients with low oocyte yields or perceived poor oocyte quality in order to purportedly maximize the chances for fertilization and, ultimately, the number of embryos to select for transfer.

Our data suggest that state mandates for ART may effectively influence clinics to use less ICSI and more eSET following recommended ASRM guidelines, compared with clinics in non-mandated states. We speculate that clinic practices in ART mandated states are in greater alignment with evidence-based ASRM practice committee guidelines. This is due in part to the ready availability of financial support for additional treatments when pregnancy and live birth is not achieved in an ART mandated state, in contrast to the “out of pocket” expenses required for future treatment in non-mandated states. This concept is further reinforced by the higher rates of eSET and lower twin birth rates per clinic and the improved clinical outcomes demonstrated through a positive correlation between the use of ICSI versus CPR and LBR in mandated states, compared to the non-mandated states over time. These data also suggest that more widespread mandates for ART insurance coverage in the United States may lead to more judicious use of ICSI as well as greater eSET, resulting in fewer complications associated with multiple births.

From 2000 and 2016, per-clinic use of ICSI increased in both the eight states with existing ART insurance mandated states and in 22 states with no mandate for insurance coverage for infertility diagnosis or treatment. However, since 2010, there has been less use of ICSI per clinic in ART mandated states compared with non-mandated states. Increased use of ICSI in non-mandated states could be associated with greater rates of MF diagnosis but it remained at a consistent level in both cohorts. Lower ICSI rates, accompanied by greater eSET rates and lower twin rates, in women < 35 years of age in ART mandated states were noted. Further studies on the use of ICSI should address the issues of semen parameters, disaggregation of embryos after conventional IVF vs ICSI in the “partial ICSI” or “ICSI split” cases and analyses that address cycle-specific confounders such as PGT cases as well as the number of transferred embryos per cycle.

## Conclusions

Starting in 2011, ICSI use per clinic developed differently among states with ART insurance mandates, compared to those without ART mandates. There was greater use of ICSI per clinic in non-mandated states, correlating with an increased frequency of MF. Lower ICSI rates per clinic accompanied by a positive correlation with CPR and LBR concurrent with a trend of greater eSET rates and lower twin rates in mandated states, suggesting that state mandates for ART coverage may encourage more selective utilization of laboratory resources.

## Supplementary information

**Additional file 1.** Appendix A. Mean Male Factor Diagnosis Rates per Clinic for fresh non-donor ART cycles with a transfer by Type of Mandate from 2000 to 2016.

## Data Availability

NASS datasets for 2000–2016 are publicly available from the CDC website: https://www.cdc.gov/art/nass/ See reference [18].

## Abbreviations

ART: Assisted reproductive technology
ASRM: American Society for Reproductive Medicine
CDC: Centers for Disease Control and Prevention
CPR: Clinical pregnancy rate
eSET: Elective single-embryo transfer
ICSI: Intracytoplasmic sperm injection
LBR: Live birth rate
MF: Male factor
NASS: National Assisted Reproductive Technology Surveillance System
PGT: Preimplantation genetic testing
