# Supplementary figures and images for: Do state insurance mandates alter ICSI utilization?
Source: Reprod Biol Endocrinol. 2020 Apr 25;18:33. doi: 10.1186/s12958-020-00589-w (PMC7183130; doi:10.1186/s12958-020-00589-w)

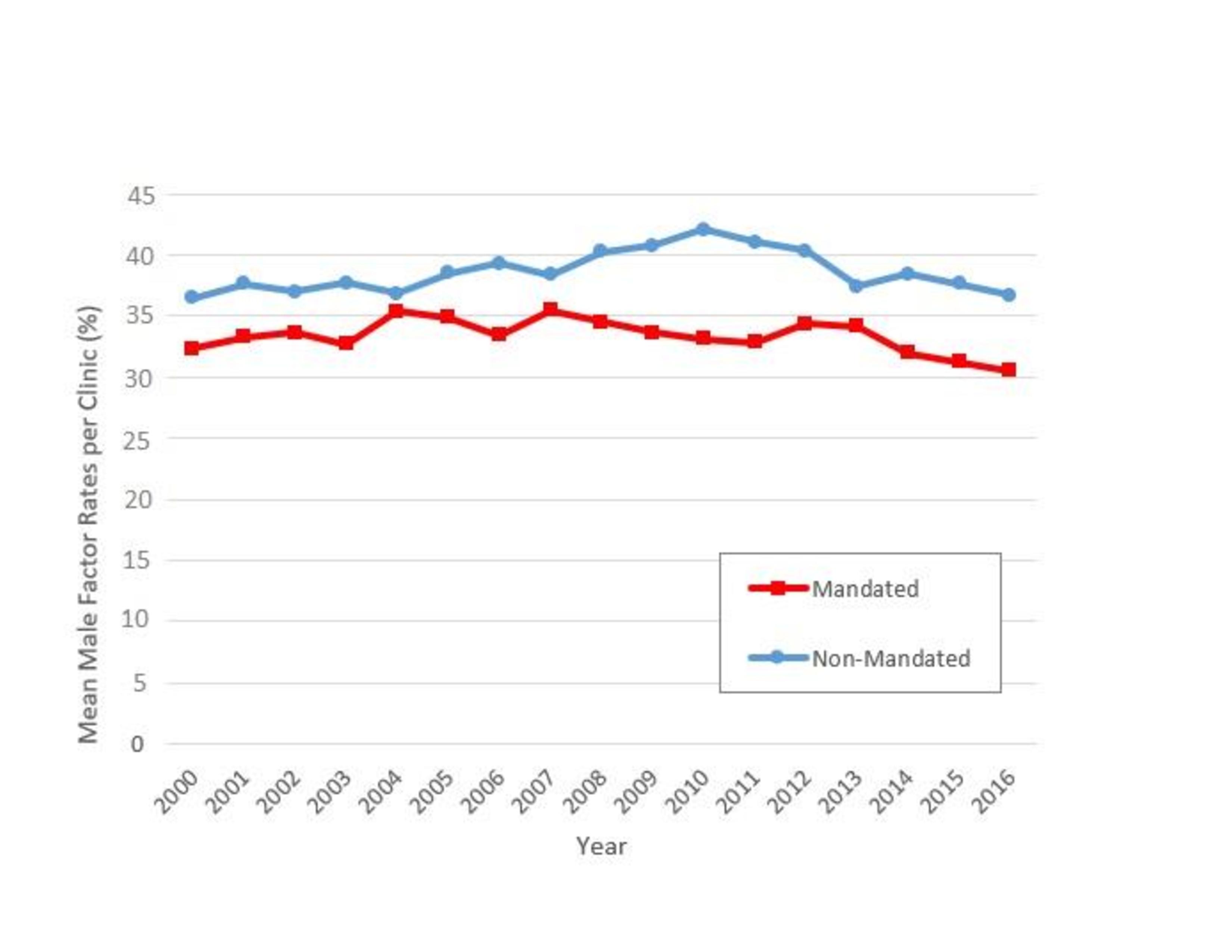

Supplement: Supplementary file 1 — Additional file 1. Appendix A. Mean Male Factor Diagnosis Rates per Clinic for fresh non-donor ART cycles with a transfer by Type of Mandate from 2000 to 2016. [file 12958_2020_589_MOESM1_ESM.jpg]
